# Copper and Trace Elements in Gallbladder form Patients with Wilson’s Disease Imaged and Determined by Synchrotron X-ray Fluorescence

**DOI:** 10.3390/jimaging7120261

**Published:** 2021-12-03

**Authors:** Wolf Osterode, Gerald Falkenberg, Fritz Wrba

**Affiliations:** 1 Universitätsklinik für Innere Medizin II, Medical University of Vienna, A-1090 Vienna, Austria; 2 Deutsches Elektronen-Synchrotron (DESY), Photon Science, D-22603 Hamburg, Germany; gerald.falkenberg@desy.de; 3 Klinisches Institut für Klinische Pathologie, Medical University of Vienna, A-1090 Vienna, Austria; fritz.wrba@meduniwien.ac.at

**Keywords:** Wilson’s disease, gallbladder, synchrotron X-ray fluorescence, copper, zinc, sulphur, metallothionein

## Abstract

Investigations about suspected tissue alterations and the role of gallbladder in Wilson’s disease (WD)—an inherited genetic disease with impaired copper metabolism—are rare. Therefore, tissue from patients with genetically characterised WD was investigated by microscopic synchrotron X-ray fluorescence (µSRXRF). For two-dimensional imaging and quantification of elements, X-ray spectra were peak-fitted, and the net peak intensities were normalised to the intensity of the incoming monochromatic beam intensity. Concentrations were calculated by fundamental parameter-based program quant and external standardisation. Copper (Cu), zinc (Zn) and iron (Fe) along with sulphur (S) and phosphorus (P) mappings could be demonstrated in a near histological resolution. All these elements were increased compared to gallbladder tissue from controls. Cu and Zn and Fe in WD-GB were mostly found to be enhanced in the epithelium. We documented a significant linear relationship with Cu, Zn and sulphur. Concentrations of Cu/Zn were roughly 1:1 while S/Cu was about 100:1, depending on the selected areas for investigation. The significant linear relationship with Cu, Zn and sulphur let us assume that metallothioneins, which are sulphur-rich proteins, are increased too. Our data let us suggest that the WD gallbladder is the first in the gastrointestinal tract to reabsorb metals to prevent oxidative damage caused by metal toxicity.

## 1. Introduction

Wilson’s disease (WD) is an inherited genetic disease with an impaired hepatic copper transport due to mutation in ATP7B which encodes the copper–transporting P-type ATPase leading to Cu accumulation in liver and extrahepatic organs [1,2,3]. Trace elements such as copper and zinc play an essential role in balancing cell metabolism. Depending on one another they are components of many metalloproteins/metalloenzymes. In particular, for copper (Cu) and zinc (Zn) a strong relationship is established in which metallothioneins (MT) play a central role [4,5,6]. The adequate excretion of elements by the liver and into the bile is of importance to maintain their appropriate homeostasis.

Since the major route for the excretion of copper and other trace elements is via the bile, it was early questioned whether the imbalance of copper metabolism in WD may affect WD gallbladder by bile [7,8]. Alterations in bile of WD have been repeatedly reported [9,10,11]. It was found that copper is associated with taurochenodexoxycholate and might be responsible for the elevated tissue copper levels of gallbladder in WD. Moreover, acute gallbladder hydrops or cholecystolithiasis have been also described in WD and have been assumed to be causative for an unbalanced Cu metabolism [12,13,14,15,16].

To image trace elements in human tissues varied methods such as e.g., laser ablation-inductively coupled plasma mass spectrometry [17] or electron probe microanalyser with wavelength dispersive spectrometers [18] have been used, whereby micro Synchrotron X-ray fluorescence (µSRXRF)—not a tissue destructing method—has been proven to be a feasible method in imaging and determining trace elements in tissues [19,20,21,22].

Since we are not aware of any study that two-dimensionally and quantitatively investigated trace elements along with phosphorus (P) and sulphur (S) in gallbladder and associated liver from WD patients we questioned whether gallbladder tissue is affected by the imbalance of copper and presumable other transient elements e.g., iron or zinc in WD using µSRXRF.

## 2. Materials and Methods

### 2.1. Control Gallbladder (C-GB)

Six archival in paraffin embedded gallbladder tissue with about 3 × 2.5 × 1.2 cm^3^ of size were available (C-Gb). Specimens were histologically rated as essentially unremarkable GB tissues. Patients at time of chirurgic removal of the gallbladder were 46 ± 6 years of age. Demographic and histological data for these C-GB specimens are summarised in Table 1.

### 2.2. Wilson’s Disease Gallbladder (WD-GB)

Gallbladder tissue from 6 patients (32 ± 11 years of age) with Wilson’s disease was investigated. Wilson’s disease was documented due to mutation in ATP7B, and the patients had to undergo liver transplantation due to severe—mostly cirrhotic—WD. Paraffin embedded tissues blocks were of about 3 × 3 × 1.5 cm^3^ of size. Table 2 summarises the demographic and histological data for the six investigated WD-GB sections.

### 2.3. Tissue Preparation for µ-SRXRF Investigations

Sections of 10 µm of thickness were cut from the paraffin embedded tissues by a metal-free microtome knife and mounted by adhesion on a trace element free Ultralene Kapton foil^®^ 4 μm of thickness. These parallel tissue sections were about 20–30 μm apart from those used for histological comparisons.

### 2.4. Microscopic Synchrotron Radiation X-ray Fluorescence Analysis

Since the technical setup was nearly identical with the one used for the investigations of WD liver [20] and which moreover was extensively described in [23], we summarise the conditions briefly. All microscopic synchrotron radiation X-ray fluorescence (μ-SRXRF) measurements were done at beamline L at the DORIS III storage ring at HASYLAB/DESY in Hamburg. The white beam of a bending magnet was monochromatised at 17 keV by a Multilayer monochromator and focussed to a spot of 15 × 15 µm^2^ by a polycapillary half-lens. The photon flux was about 10^11^ photons/s. Measurements were done at room temperature and in air environment. Tissue sections mounted on a XYZ-sample stage were raster scanned with an increment of 15 µm in horizontal and 15 µm in vertical direction, respectively at a sample time of 1.25 s per investigated scan point.

**Figure 1:**
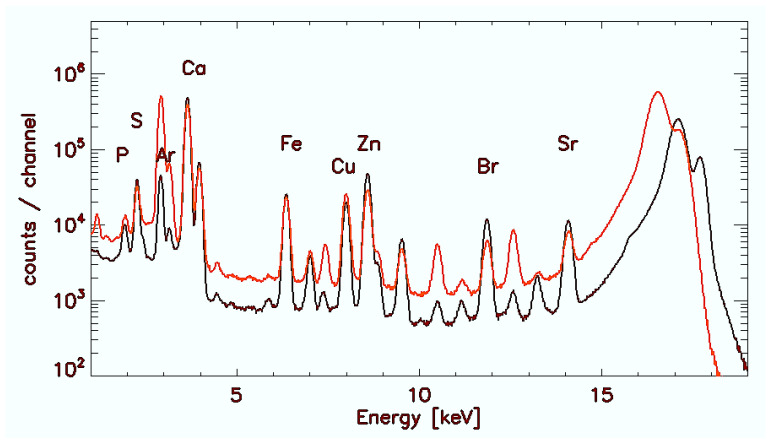
Accumulated spectra after summation over all pixels with peaks of the investigated elements. Red colour: Control–GB. Black colour: Wilson’s Disease-GB.

In counts/channel Figure 1 demonstrates exemplarily accumulated spectra after summation over all scan points in C-GB (red) and WD-GB (black) with characteristic elemental peaks.

### 2.5. Quantification of Elements

As described before each individual spectrum was peak-fitted by the AXIL program package [24]. The net intensities of fluorescence lines were normalised to the primary beam intensity. A germanium reference foil of homogeneous Ge area density of 2.6 × 10^−7^ g/cm^3^ (3.4% standard deviation) was used for external standardisation. The Ge reference film was generated by sputtering pure germanium (purity 99.999%) on a polycarbonate foil of 2 µm thickness [25]. Element concentrations of the sample were calculated from elemental peak intensities applying the fundamental parameter-based program package ‘quant’. Since the sample can be considered as a single layer thin film of known thickness for all detected elements but P and S, the calculation can be purely based on theoretical equations and a fundamental parameter database with normalisation in response to the known germanium reference. Only mean concentration values within the measurement voxel of 15 × 15 × 10 μm^3^ can be given. The voxel extends through the complete thickness of the thin sample section which allows a simple conversion of areal densities into (mean) concentrations. For the light elements P and S the given concentration, values should be considered as qualitative only.

### 2.6. Statistical Analysis

Mean and standard deviations were calculated, and Student’s t-test was applied for comparison. Results are reported as mean ± SD. A value of *p* < 0.05 was considered to be statistically significant. Relationship between elements are tested by the nonparametric Spearmen’s–Rank correlation. Spearmen’s–Rank correlation r_s_ measures the strength and direction of association between two ranked variables (SigmaStat© 3.5).

## 3. Results

### 3.1. Control Gallbladder (C-GB)

**Figure 2:**
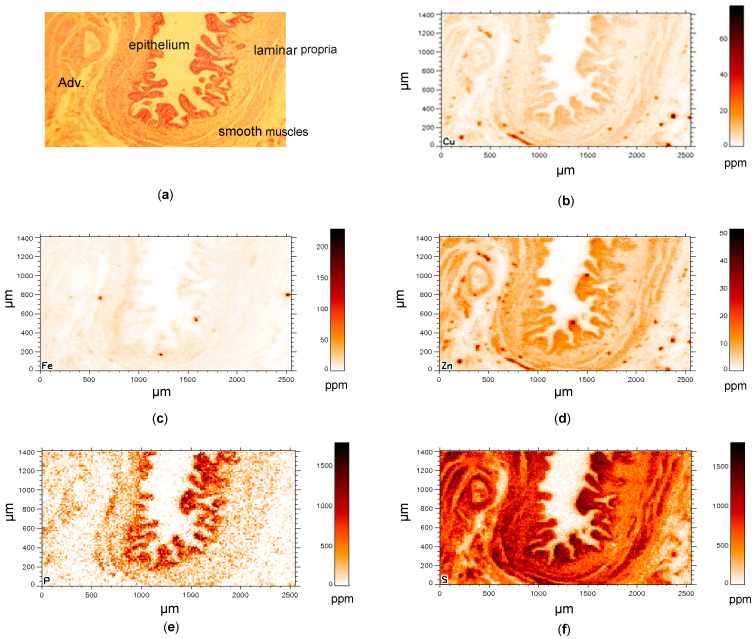
(**a**–**f**) Element distribution in a control gallbladder. (**a**) Microscope recording of the investigated gallbladder tissue with epithelium (columnar cells), beneath the epithelium the laminar propria (connective tissue) and smooth muscular fibres for contraction. Adventitia (Adv): perimuscular connective tissue that is very dense and connects with the liver. (**b**–**f**) copper (Cu), iron (Fe), zinc (Zn), phosphorous (P) and sulphur (S) distribution. The histological section is about 30-µm apart from the investigated one for SRXRF.

**Figure 3:**
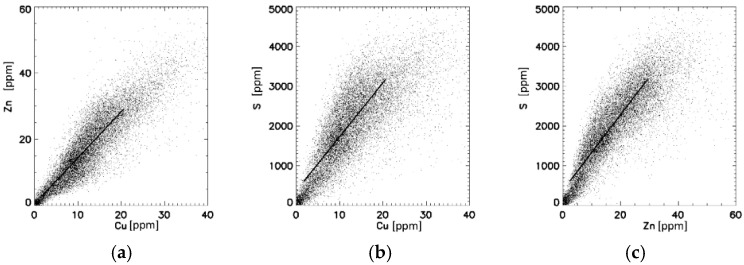
Correlations between elements in ppm for C-GB (Figure 2). (**a**) Cu:Zn = 1.28; r_s_ = 0.92. (**b**) S:Cu = 70; r_s_ = 0.92. (**c**) Correlations between S:Zn = 79, r_s_ = 0.88. For all *p* < 0.001.

Demographic and histological data are shown for control gallbladder (C-GB) tissues in Table 1. From the histological point of view, only minor pathological abnormalities existed. Figure 2 exemplarily shows the distribution of different elements in a C-GB in comparison with the microscope image of the histological section (Figure 2a) that was about at a distance of 20–30 µm apart from the µSRXRF-investigated section. Histological features like epithelium, laminar propria, smooth muscles and adventitia with vessels (Figure 2a) are very similar resolved in the µSRXRF elemental images (Figure 2b–f). In all scans, Cu and Zn like S were mostly found in epithelium and smooth muscles. P predominantly characterises epithelium. Altogether, more than 200,000 single measurements (i.e., analysed single spectra) were used for elemental distribution resulting in a near histological image (Figure 2b–f). Obvious associations between elements in C-GB are given for Cu/S, Cu/Zn and Zn/S as documented in Figure 3a–c. Significant correlations were calculated for Cu/Zn with r_s_ = 0.92 or r_s_ = 0.88. For Cu/S with r_s_ = 0.92 or r_s_ = 0.75, and Zn/S with r_s_= 0.88 or r_s_ = 0.82.

### 3.2. Wilson’s Disease Gallbladder (WD-GB)

For comparison, element concentrations in C-GB and in WD-GB are shown in Table 3, demonstrating that all investigated elements were found to be increased in WD gallbladder.

**Figure 4:**
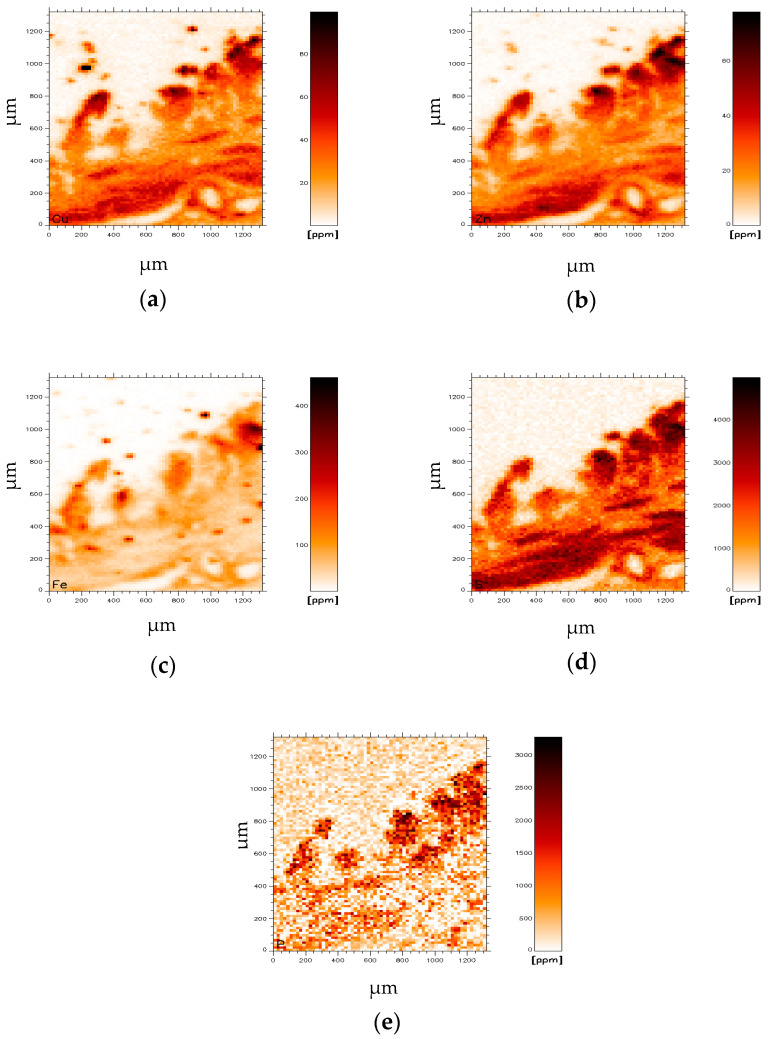
Distribution of elements in a WD gallbladder. (**a**) copper (Cu), (**b**) zinc (Zn), (**c**) iron (Fe), (**d**) sulphur (S) and (**e**) phosphorus (P).

Element imaging of WD-Gb tissue reveals high Cu and Zn concentrations in the area of presumable GB epithelium and in smooth muscles (Figure 4a,b). The distribution of elements, also Cu, Zn and P (Figure 4c–e), in this sample are very similar.

**Figure 5:**
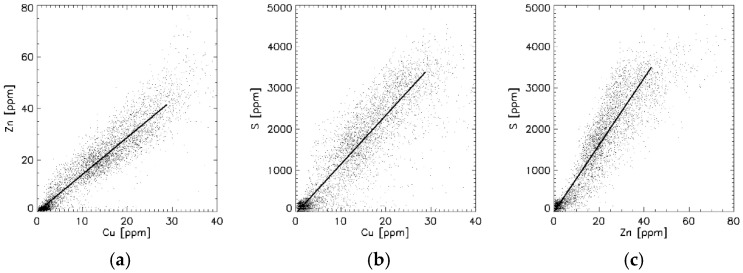
Correlations between elements for a WD-GB. (**a**) Cu/Zn = 1.28; r_s_ = 0.94. (**b**) S/Cu = 119; r_s_ = 0.91. (**c**) Correlations between S/Zn = 81, r_s_ = 0.93. For all *p* < 0.001.

To demonstrate associations between elements, scattergrams were drawn and associations were calculated, showing significant correlations between Cu/Zn (Figure 2b), Cu/S (Figure 2c) and Zn/S. Correlations between elements are shown in Figure 5.

To compare elemental levels with those of the corresponding WD livers (GB and liver were for these cases surgically together removed due to the severity of the WD illness) data are given in Table 4. Data resemble nearly those found in 12 WD patients previously investigated [20]. In contrast to WD-liver mean P and S were decreased while Fe and Zn were unaffected. Only Cu in WD liver was exorbitantly increased.

## 4. Discussion

In this report we used micro synchrotron X-ray fluorescence (µSRXRF) to image and analyse elements in gallbladder from patients with genetically documented Wilson’s disease in which Cu overload in liver cells are characteristic for their impaired hepatic copper transport due to mutation in ATP7B [1,2,3]. Since µSRXRF simultaneously allows the detection of different elements, their two-dimensional maps facilitate comparisons with histological structures as seen in microscope recordings and may in addition be used for patho/physiological interpretations. We showed that mean concentrations of all determined elements in WD gallbladder (WD-GB) tissue were increased. This is in contrast to prior results in WD-liver [20] in which P and S were lower while Fe and Zn remained unaffected. As expected only Cu in WD liver was increased and exorbitantly in hepatocytes. Data in Table 3 belong to six WD-livers explanted together with the WD-GBs due to severe liver failure.

Under physiological conditions plasmatic hepatic tissue removes Cu from the circulation by rapidly trapping the metal in chelating Cu proteins, whereas Cu excess is excreted in bile and into the gallbladder [26,27]. Bile is a major route for the excretion of heavy metals [27]. The gallbladder lumen is covered by tall columnar epithelial cells, which are specialised for absorption. Although they are mostly adapted for salt and water resorption [28] we assume that they thereby incorporate metals like Cu and Zn and Fe from the bile. In particular a strong relationship between copper and Zn has been repeatedly shown in Wilson’s disease, a finding that was also documented in animal models of WD [29,30,31]. Therefore, zinc acetate was applied for the treatment of WD. Zinc in excess upregulates copper-binding metallothionein in the gut mucosa, leading to increased elimination of copper and negative Cu balance [32,33]. Both copper and zinc are bound to various proteins, but they are preferentially bound to MT [4,34]. Metallothioneins are sulphur-rich proteins. Therefore, sulphur concentrations were also considered in this investigation, assuming sulphur can indirectly mimic MT. A significant linear relationship between Cu, Zn and S could be demonstrated in Figure 3a–c. The striking correlations between Cu/S and Zn/S, may underline their relationship to metallotionein [27,35].

Depending on the investigated tissue regions, calculated ratios of Cu/Zn ranged between 0.89 and 1.28 in C-GB with correlations coefficients r_s_ between 0.7 and 0.9. Although the affinity for copper to MTs is greater than for Zn, the Cu/Zn ratio in WD-GB was nearly identical (Table 3). We may assume that the relatively small increase in Cu and Zn in WG-GB was compensated by the MT buffer capacity since S was likewise increased, speculating that the increased sulphur/MT is caused to detoxify accumulated metals or to prevent oxidative damage caused by metal toxicity [36,37,38].

Concerning Fe, there are no data about its tissue content in WD-GB. Studies of copper proteins (ceruloplasmin and hephastin) have provided links connecting the pathways of copper and iron [26,29,39,40]. Clinical results documented that in WD patients an increased intestinal mucosal Fe concentration existed which was similar in controls with duodenitis [37]. This seems to be plausible. Reabsorbing similarities between GB and small intestine epithelium do exist [41] and an enhanced Fe would be in line with our finding in WD-GB.

As a side aspect in this context, we may mention that focal iron accumulation in the brain plays a crucial role in neurodegenerative diseases [42,43]. In particular, in Wilson’s disease, Fe-accumulation in the putamen or nucleus dentatus in parallel to Cu increase is described [44,45,46]. The metabolism and the interplay between iron and copper in this genetic disease depends on various causative genes. Up to 15 genes have been identified to date [42,43]. As stated earlier, epithelial cells of the GB are specialised for absorption and thus quite different to neuronal cells. We may assume that the high Fe-absorption rate of the Gb and small intestine promotes the (focal) cellular uptake in specialised brain areas.

In the upper small intestine phosphorus (P) is predominantly absorbed as inorganic phosphate which is transported into the epithelial cells by cotransport with sodium. Expression of this (or these) transporters is promoted by vitamin D [28]. It may be argued, since both the endothelium of GB and small intestine are composed of large cuboidal cells with in parts similar functional properties, that the nearly exclusive P distribution in the epithelium of GB-e.g., seen in Figure 2e and Figure 4e is the result of inorganic P absorption.

The principal limitations of this study are that only six genetically characterised WD-GB were accessible, and the tissue was only available as embedded in paraffin-blocks. That means they were fixed in 4% buffered formalin for at least 24 h, dehydrated in ethanol and ethanol cleared with xylene before waxing. Such procedure alters elemental tissue concentrations. Previous investigations at the same beamline comparing tissue fixation by formalin or paraformaldehyde with paraffin-embedded brain sections observed lower concentrations of most elements [47]. Paired brain specimens one conserved by formalin while the other was rapidly frozen, showed a decrease of iron and zinc with fixation. Iron was reduced by 40% (*p* < 0.01), and zinc by 77% (*p* < 0.0001), but Cu concentrations increased by 37%. The increase in copper is likely due to contamination from trace copper in the formalin [48]. Nevertheless, Chwiej et al. [47] stated that comparison between analogous areas of two independent samples, as sample and control in our case, seems valid. In own studies on paired liver tissue (shock-frozen/paraffin) we found chloride, potassium increased while Cu in paraffin sections was 20–30% increased and Fe and Zn only 10–15% reduced (personal communication W.O. and G.F). Slight differences may depend on the tissue type. Shock-frozen-hydrated tissue probes are now the generally accepted and recommended method to preserve the chemical and structural integrity of the cells [49,50,51].

However, the paraffin embedded tissue of the GB-C and GB-WD was processed by the same method and on the same tissue type so that the processes of partially washing out of their elements were similar. The main interest in this study was focused on Cu, Zn and Fe, metals that are normally bound to proteins in tissue. Thus, we may assume that on the tissue level their distribution is maintained. Another limitation of the study may be that the access to SRXRF devices is rare.

## 5. Conclusions

In comparing histological sections with the bio-metal imaging by µSRXRF and additional quantification of elements, we could show that Cu and Zn and Fe are predominantly increased in the epithelium of WD-Gallbladder. These concentrations are relatively low compared to those in WD hepatocytes. The significant linear relationship with Cu, Zn and sulphur let us assume that metallothioneins, which are sulphur-rich proteins, are increased too. We suggest that GB is the first in the intestinal tract to reabsorb metals to prevent oxidative damage caused by metal toxicity.

## Figures and Tables

**Table 1:** Demographic data of control gallbladder tissue (C-GB). Age [years] at the time of chirurgical removal. FDG: signs of fatty degeneration, SC: signs of cholestasis and SoI: signs of inflammation.

No.	Age [Years]	Gender	Tissue	Histology		
				FDG	SC	Sol
1	51	m	Block	no	±	no
2	40	m	Block	no	no	no
3	36	f	Block	no	no	?
4	48	f	Block	±	no	no
5	53	m	Block	no	no	no
6	46	f	Block	no	no	±

**Table 2:** Demographic data of WD gallbladder tissue (C-GB). Age [years] at the time of chirurgical removal of liver and gallbladder. FDG: signs of fatty degeneration, SC: signs of cholestasis and SoI: signs of inflammation.

No.	Age [Years]	Gender	Tissue	Histology		
				FDG	SC	Sol
1	29	f	Block	no	±	±
2	40	m	Block	+	no	?
3	18	f	Block	no	no	?
4	32	f	Block	±	no	no
5	48	m	Block	no	no	no
6	25	f	Block	±	+	±

**Table 3:** Mean element concentrations in ppm ± SD in control gallbladder (C-GB) and WD-GB (N = 6). Significant differences are indicated by * for *p* < 0.05, or ** for *p* < 0.01.

N = 6	P	S	Cu	Fe	Zn	
C-GB	849	1250	10	8	12	[ppm]
SD	261	148	4	4	3	[ppm]
WD-GB	1796 *	2350 **	23 **	67 *	30 **	[ppm]
SD	912	651	9	28	11	[ppm]

**Table 4:** Mean element concentrations in ppm ± SD in control liver (C-L) and WD-L. For WD-L data were stratified into fibrotic areas (Fibr.) and hepatocytes (Hep.). Significant differences between C-L and WD-L, (fibrotic area and hepatocytes, respectively) are indicated by * for *p* < 0.05, or by ** for *p* < 0.01.

N = 6		P	S	Cu	Fe	Zn
C-L		1818	2699	20	299	64
SD		497	457	5	34	19
WD-L	Fibr.	1269 *	1687 **	83 **	293	55
	SD	319	275	14	99	12
	Hep.	1301	2121 *	434 **	381	71
	SD	401	369	107	151	12

## Data Availability

Not applicable.

## Abbreviations

The following abbreviations are used in this manuscript:

WD	Wilson’s disease
GB	Gallbladder
SRXRF	Synchrotron X-ray fluorescence
MT	Metallothionein
